# Targeting cell cycle in leukemia: “Is palbociclib the game-changer?”: A review

**DOI:** 10.1097/MD.0000000000046873

**Published:** 2026-01-02

**Authors:** Muhamad Amir Azizan, Zainul Abeden, Nur Haida Natasha Shamsuddin, Fadly Ahid, Narazah Mohd Yusoff, Mohd Nazri Ismail, Siew Kit Ng, Asmida Isa

**Affiliations:** a Department of Biomedical Science, Advanced Medical and Dental Institute, Universiti Sains Malaysia, Kepala Batas, Pulau Pinang, Malaysia; b Centre for Medical Laboratory Technology Studies, Faculty of Health Sciences, Universiti Teknologi MARA, Puncak Alam, Selangor, Malaysia; c Clinical Diagnostic Laboratory, Pusat Perubatan USM Bertam, Advanced Medical and Dental Institute, Universiti Sains Malaysia, Kepala Batas, Pulau Pinang, Malaysia; d Analytical Biochemistry Research Centre, Universiti Sains Malaysia, Bayan Lepas, Penang, Malaysia.

**Keywords:** ALL, AML, CDK4/6 inhibitor, CDK6, cell cycle, leukemia, palbociclib

## Abstract

Leukemia is a complex and heterogeneous disease, making it challenging to determine the correct treatment. Over the past few decades, there have been few changes in standard medical care. To mitigate this problem, several potential small molecules that target the disease at the molecular level are being investigated for their clinical significance in leukemia treatment. Among them, the cyclin-dependent kinase (CDK) inhibitor palbociclib emerges as a promising therapeutic candidate targeting CDK4/6. Although palbociclib was approved by the Food and Drug Administration for the treatment of HER2-negative and HR-positive advanced or metastatic breast cancer, its potential in leukemia is still under research. Furthermore, CDK6 has been discovered to have essential roles in leukemic cells beyond its involvement in cell cycle progression, making it a notable target in leukemia. This review comprehensively analyses existing literature on the effectiveness and safety of using palbociclib alone or in combination with other treatments in preclinical and clinical leukemia investigations.

## 1. Introduction

Leukemia, characterized by the clonal expansion of abnormal blood cells within the bone marrow, is a significant global health concern, with 2.5% of all new cancer cases and 3.1% of cancer-related deaths as of 2020.^[1]^ The primary treatment options for leukemia are chemotherapy and hematopoietic stem cell transplantation. Despite recent advances in targeted therapies and immunotherapies, the 5-year relative survival rates for leukemia are only 67% and dropped lower in acute myeloid leukemia (AML) to approximately 31.9%.^[2,3]^ Diagnosing and treating leukemia remains a challenge due to the disease’s heterogeneity, resistance development, and high toxicity of the current treatment.^[4]^ Thus, there is a need for a more specific and safe treatment to address these problems.

**Figure 1.**
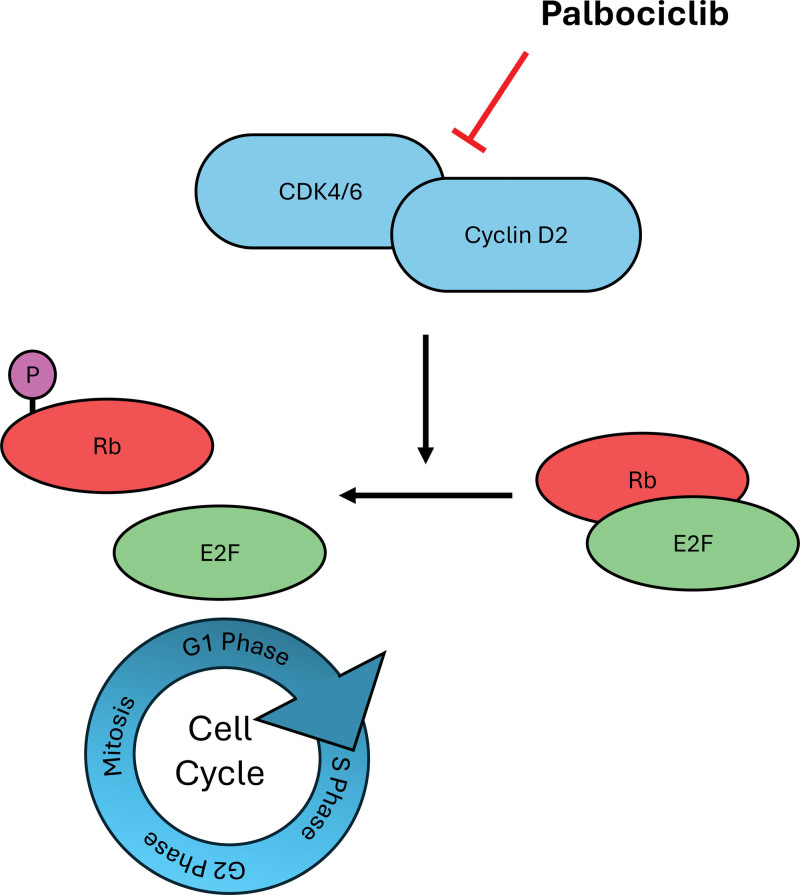
Mechanism of action of palbociclib. Palbociclib selectively inhibits the kinase activity of the CDK4/6–CCND2 complex, resulting Rb to remain in its hypophosphorylated, active state. Active Rb suppresses E2F transcription factor activity, which can lead to G1 cell cycle arrest. CDK4/6 = cyclin-dependent kinases 4/6; E2F = E2 promoter-binding factor; P = phosphate group; Rb = retinoblastoma protein.

Palbociclib, the specific inhibitor for cyclin-dependent kinase 4/6 (CDK4/6), emerged as a promising therapeutic agent to treat various cancers.^[5,6]^ Palbociclib inhibits cell proliferation by blocking the kinase activity of the cyclin D–CDK4/6 complex, resulting in hypophosphorylation of retinoblastoma (Rb), and subsequently, deactivation of the E2F transcription factor that leads to cell cycle arrest at the G1 phase (Fig. 1). Palbociclib has been used together with an aromatase inhibitor or with fulvestrant, an estrogen receptor antagonist.^[7,8]^ This small molecule inhibitor was developed by Pfizer Inc. and authorized by the Food and Drug Administration in March 2015 upon demonstrating significant improvement in progression-free survival in women with advanced estrogen receptor (HR)-positive and human epidermal growth factor receptor-negative (HER2-negative) breast cancer.^[9]^ While currently authorized for the treatment of HR-positive and HER2-negative advanced or metastatic breast cancer, palbociclib’s potential as treatment in other cancers, including leukemia, is still under research. Therefore, this review aims to discuss the current literature on using palbociclib in leukemia and evaluate its mechanism, efficacy, safety, and potential as a therapeutic option for leukemia.

## 2. Role of CDK4 and CDK6 in leukemia

CDK4 and CDK6 play critical roles, particularly in the G1/S checkpoint. CDK4/6 activation by cyclin D, triggered by mitogenic signals, sets the stage for cell cycle progression. The cyclin D-CDK4/6 complex phosphorylates Rb, which releases the E2F transcription factor. This factor is critical for the transcription of proteins required for the cell cycle to progress into the S phase. Knockout studies underline the critical role and reliance of CDK4/6 in tumor cell growth.^[10]^ Furthermore, data mining of previously published data reveals the upregulation of CDK6 mRNA levels in many subtypes of leukemia, particularly acute subtypes of leukemia, including AML, suggesting a pivotal role of CDK6 in leukemia progression.^[11–16]^ Aside from that, recent findings suggest that CDK6, but not CDK4, has additional functions as oncogenic stress regulator, hematopoietic stem cell and leukemic stem cell activator, and transcriptional regulator in Fms-like tyrosine kinase 3-internal tandem duplication (FLT3-ITD)-positive myeloid leukemia for oncogenic *FLT3* and (proto-oncogene, serine/threonine kinase 1) *PIM1.*^[17]^ In addition, In t(8;21) AML, cyclin D2, the partner for CDK4/6, is essential for leukemic propagation and was shown to be upregulated by RUNX1–ETO fusion protein.^[18,19]^ Thus, all these findings emphasize the potential of CDK4/6 as selective targets for therapeutic intervention of leukemia.

## 3. Activity of palbociclib in preclinical studies in leukemia

There are at least 3 CDK4/6 inhibitors: palbociclib, ribociclib, and abemaciclib. All 3 drugs selectively target CDK4/6 except abemaciclib, which additionally targets other CDKs as well.^[20]^ However, palbociclib is more often utilized for leukemia research. Thus, the review will focus on palbociclib activity as a stand-alone and combined treatment. The literature was sourced from PubMed database.

### 3.1. Palbociclib as a stand-alone treatment

**Figure 2.**
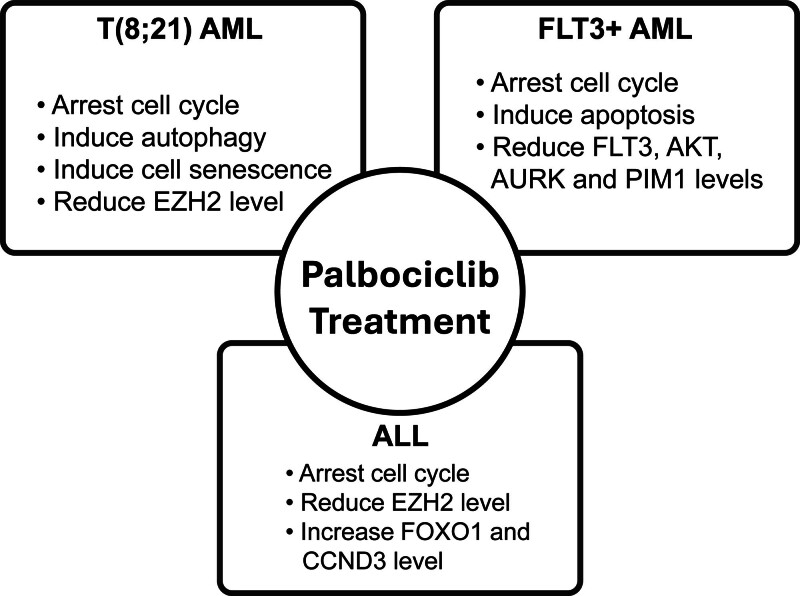
Cellular effects of palbociclib treatment in different leukemia subtypes. AKT = protein kinase B; AURK = Aurora kinase; CCND3 = Cyclin D3; EZH2 = Enhancer of Zeste Homolog 2; FLT3 = Fms-like tyrosine kinase 3; FOXO1 = Forkhead box O1; PIM1 = proto-oncogene, serine/threonine kinase 1.

Palbociclib, a potent and selective inhibitor of CDK4/6, has emerged as a compelling therapeutic intervention for treating leukemia. In vitro studies have demonstrated promising antiproliferative activities of palbociclib in both AML and acute lymphoblastic leukemia (ALL), as summarized in Table 1.^[19,21–26,28–30,33,34]^ The key cellular effects of palbociclib treatment across different leukemia subtypes are illustrated in Figure 2. In addition to impairing cell growth and slightly increasing apoptosis, palbociclib treatment caused more than a 10-fold increase in senescence-associated B-galactosidase levels, indicating the induction of cell senescence in t(8;21) AML.^[19]^ Interestingly, the treatment caused a reduction in *Enhancer of Zeste Homolog 2* transcript levels both in AML and ALL,^[19,32]^ suggesting that it is unlikely to promote cellular quiescence.^[35]^

**Table 1:** Palbociclib treatment effect on leukemia.

Leukemia type	Palbociclib dosage	Treatment effect	Reference
AML	100 nM, 300 nM, and 1000 nM	In vitro: • Induce cell cycle arrest at G1 phase with slight apoptosis (OCI-AML3) • Induce cell cycle arrest at G1 phase and apoptosis (MV4-11)	Ling et al^[21]^
	500 nM	In vitro: • Induce G1 cell cycle arrest. • Decrease *HOXA9* and *PIM1* mRNA level.	Yang et al^[22]^
AML (t(8;21))	75 mg/kg	In vivo: • Induce autophagy. • Suppress tumor growth.	Matsuo et al^[23]^
	500 nM	In vitro: • Induce G1 cell cycle arrest. • Induce autophagy.	Nakatani et al^[24]^
	Cell lines: 50 nM Primary cells: 300 nM	In vitro: • Induce G1 cell cycle arrest with slight apoptosis. • Induce cell senescence. • Inhibit colony formation. • Reduce *EZH2* transcript level.	Martinez-Soria et al^[19]^
	100 mg/kg	In vivo: • Decrease leukemic burden. • Delayed AML progression. • Increase overall survival.	
AML (MLL-r)	500 nM	In vitro: • Induce G1 cell cycle arrest.	Matsuo et al^[23]^
	300 nM and 1000 nM	In vitro: • Inhibit colony formation in immortalized and primary cell lines. • Induce cell differentiation	Placke et al^[14]^
AML (FLT3-ITD)	500 nM	In vitro: • Induce G1 cell cycle arrest. • Inhibit colony formation. • Does not induce apoptosis.	Lopez et al^[25]^
	1000 nM	In vitro: • Induce G1 cell cycle arrest. • Induce apoptosis. • Decrease *FLT3* mRNA and protein level and autophosphorylation. • Decrease *PIM1* mRNA level.	Uras et al^[26]^
	25 mg/kg	In vivo: • Block tumor formation and growth. • Increase overall survival	
	500 nM	In vitro: • Induce G1 cell cycle arrest in cell lines and primary blast. • Sub-G1 peaks at 120 hours indicating slight apoptosis.	Wang et al^[27]^
	150 mg/kg	In vivo: • Decrease growth and proliferation. • Increase overall survival.	
AML (FLT3-ITD and FLT3-TKD)	300 nM 15 mg/kg	In vitro: • Impair viability and colony formation. • Induce apoptosis. • Reduce expression of *Akt* and *AURK* mRNA In vivo: • Block tumor formation and growth	Uras et al^[28]^
B-ALL	0.5–4.0 µM 75 mg/kg	In vitro: • Induce G1 cell cycle arrest. In vivo: • Inhibit tumor growth and proliferation. • Increase overall survival.	Bride et al^[29]^
	1 µM	In vitro: • Induce G1 cell cycle arrest. • Does not induce significant apoptosis • Increase FOXO1 protein expression • Increase *Ccnd3* mRNA and protein expression	Ketzer et al^[30]^
T-ALL	75 mg/kg	In vivo: • Decreased the number of human CD45 + (hCD45+) cells. • Increase overall survival	Bride et al^[29]^
Ph + Lymphoid Leukemia	100 nM	In vitro: • Induce G1 cell cycle arrest. • Induce apoptosis in 2 cell lines (KOPN72b and SU-Ph2).	Nemoto et al^[31]^
	150 mg/kg	In vivo: • Blocked rapid dissemination of leukemic cells in bone marrow and blood. • Prolonged Survival: SU-Ph2 (16 days) and KOPN72b (13 days).	
ALL (MLL-r)	1 µM	In vitro: • Induce G1 cell cycle arrest. • Reduce EZH2 expression.	Linden et al^[32]^

µM = micromolar, Akt = protein kinase B, AML = acute myeloid leukemia, AURK = aurora kinase, B-ALL = B-cell acute lymphoblastic leukemia, CCND3 = Cyclin D3 gene, EZH2 = Enhancer of Zeste Homolog 2, FLT3 = FMS-like tyrosine kinase 3, FOXO1 = Forkhead box O1 gene, G1 = G1 phase of the cell cycle, HOXA9 = homeobox A9 gene, mg/kg = milligrams per kilogram, MLL-r = MLL-rearranged, nM = nanomolar, PIM1 = proto-oncogene, serine/threonine kinase 1, T-ALL = T-cell acute lymphoblastic leukemia.

Conversely, Nakatani et al found that CDK4/6 inhibition using palbociclib in t(8;21) AML induced autophagy and an increase in the number of autophagosomes compared to the control sample.^[24]^ This is further confirmed in the t(8;21) AML mouse xenograft model.^[23]^ Autophagy is an adaption mechanism that recycles amino acids by breaking down defective cellular components under conditions of cellular stress, such as nutrient deprivation or drug treatment.^[36]^ Studies have demonstrated that autophagy serves as an attempt by AML cells to resist the effects of palbociclib treatments, suggesting that a combination of palbociclib and autophagy inhibitors could prove to be effective in treating AML.

As shown in several studies, palbociclib also effectively inhibits the proliferation of FLT3-ITD AML.^[25,26,28,34]^ The FLT3 mutation is one of the most common mutations in AML, occurring in approximately 30% of AML cases and associated with a poor prognosis, making it one of the most critical targets for AML treatment.^[37]^ The antiproliferative action is caused specifically by its CDK6 inhibition, as knockdown studies demonstrate that CDK4 is dispensable in FLT3-ITD AML.^[25]^ When the palbociclib dose was doubled to 1 μM, a 5-fold increase in the sub-G1 compartment was observed, indicating an induction apoptosis in FLT3-ITD AML.^[26]^

Moreover, palbociclib treatment in FLT3-ITD AML decreases the mRNA expression for FLT3 and PIM1.^[28]^ FLT3 kinase activity activates the signal transducer and activator of transcription 5, phosphoinositide 3-kinase/protein kinase B (PI3K/AKT), and mitogen-activated protein kinase signaling pathways and plays a dominant role in cell survival, proliferation, and differentiation.^[38]^ Meanwhile, PIM1, a downstream of FLT3-ITD, is essential for leukemia proliferation and its anti-apoptotic effect. Chromatin immunoprecipitation assays have revealed that CDK6 is enriched at the *FLT3* and *PIM1* promoters and regulates the expression of FLT3 and PIM1 proteins.^[28]^ Furthermore, the FLT3/HCK/CDK6 pathway led to the overexpression of CDK6 in FLT3-ITD AML cells, indicating a vicious cycle that enhances the survival and progression of leukemic cells.^[25]^ Palbociclib targets not only the cell cycle but also FLT3 and PIM1 in FLT3-ITD, indicating its potential for use alone or in combination with FLT3 or PIM1 inhibitors in treating this subtype of leukemia.

Meanwhile, in cell lines with wild-type FLT3 (THP-1 and U937), it was discovered that CDK2 could compensate for the inhibited CDK4/6 activity caused by palbociclib.^[34]^ After 5 days of prolonged palbociclib treatment, partial phosphorylation of Rb was detected. Downregulation of p27^Kip1^ (CDK2 natural inhibitors) and upregulation of Cyclin E, especially noted in U937 cells, facilitated this reentry into the cell cycle, explaining the tolerance of wt-FLT3 cells. These changes were not observed in FLT3-ITD AML (MOLM13 and MV4-11), and the Rb maintains its dephosphorylated state showing that palbociclib is not as effective in cell lines with wild-type FLT3.

Palbociclib also induces cell cycle arrest in ALL cell lines, but no significant apoptotic activity was observed. However, in Philadelphia chromosome (Ph)-positive ALL, a 120-hour-long palbociclib treatment has been shown to induce apoptotic activity in 2 of the tested cell lines.^[31]^

In vivo studies have also corroborated the effectiveness of palbociclib as an antiproliferative agent. In both AML and ALL, palbociclib decreased tumor burden and leukemic growth, leading to increased overall survival.^[19,26,28,29,31,34]^

### 3.2. Palbociclib in combinations with other treatment agents

Palbociclib has been used with other leukemia treatments such as chemotherapy, kinase inhibitors, and others to treat leukemia (Table 2). Most of these combinations have displayed a remarkable efficacy and synergistic effect in preclinical studies. These led to a better understanding of therapeutic approaches for leukemia, which could address the challenges associated with treatment resistance and toxicity in leukemia.

**Table 2:** Palbociclib in combination with other drugs in leukemia.

Agents	Leukemia type & dosage		Treatment effect	Synergism	Reference
Palbociclib + chemotherapy					
Palbociclib + Doxorubicin + Cytarabine	AML OCI-AML3 [Pal (100–1000 nM) + Dox/AraC (8/320 nM)] MV4-11 [Pal (100–1000 nM) + Dox/AraC (2/80 nM)]		In vitro: • Increase apoptotic activity.	Bliss score was either addictive or synergistic across all cell lines evaluated	Ling et al^[21]^
	NA		In vivo: • Moderately reduced terminal peripheral blood GFP and reduced spleen size in vivo		
Palbociclib + Cytarabine	AML Pal (0.5 or 1 µM) + AraC (1 or 5 µM)		In vitro: • Sequential treatment enhances cytotoxicity in both cell lines and primary cells.	NA	Yang et al^[22]^
	Pal (150 mg/kg) + AraC (1.6 mg/kg or 10 mg/kg)		In vivo: • Reduce tumor load by 16 times. • Better survival than other group assessed (no death until the end of experiment).	NA	
Palbociclib + Dexamethasone	ALL Pal (150 mg/kg) + Dex (2 mg/kg)		In vivo: • Both sequential and simultaneous treatment enhance cytotoxic effect	NA	Bride et al^[29]^
Palbociclib + L-asparaginase	ALL Pal (150 mg/kg) + L-asp (0.1 mg/kg)		In vivo: • Sequential treatment has no effect. • Simultaneous treatment enhances the cytotoxic effect.	NA	Bride et al^[29]^
Palbociclib + Vincristine/ Vinblastine	ALL Pal (150 mg/kg) + VCR (5000 IU/kg)		In vivo: • Sequential treatment has no effect. • Simultaneous treatment enhances the cytotoxic effect. • Increase overall survival in T-ALL (Cul76)	NA	Bride et al^[29]^
	ALL Pal (1 µM) + VCR (0.1 or 1 µM)		In vitro: • Induce resistance to Vincristine	NA	Delgado et al^[39]^
	ALL Pal (100 nM) + VCR (1.5 ng/ml)		In vitro: • Induce resistance to Vincristine	NA	Nemoto et al^[31]^
Palbociclib + FLT3 inhibitors					
Palbociclib + Quizartinib	AML (FLT3^+^)		In vitro: • Inhibit cell proliferation (IC50). • No sign of resistance observed when treated with low concentration (IC30). • Decrease expression of FLT3 and CDK4 • Inhibit phosphorylation of AKT	CI: synergistic	Yang et al^[40]^
Palbociclib + Gliteritinib	AML (FLT3+)		In vitro: • Inhibit cell proliferation (IC50). • No sign of resistance when treated with low concentration (IC30)	CI: synergistic	Yang et al^[40]^
Palbociclib + TCS-359/ Tandutinib/ Quizartinib	AML (FLT3-ITD) Pal (1 µM) + TCS-359 (0.3 µM)		In vitro: • Impair cells lines and primary cell growth. • Increase in apoptosis (Cell accumulates in sub-G1 phase).	CI: synergistic	Uras et al^[26]^
Palbociclib + SU14813	AML (FLT3-ITD) Pal (500 nM) + SU14813 (30 nM or 100 nM)		In vitro: • Pal enhances the pro-apoptotic effect of SU14813 in cell lines. • Combination has no effect on primary cells.	CI: synergistic	Wang et al^[34]^
Palbociclib + PI3K/mTOR/AKT inhibitors					
Palbociclib + Copansilib		AML (FLT3+)	In vitro: • Decrease cell proliferation. • No sign of resistance. • Reduce CDK4 expression. • Inhibit phosphorylation of AKT	Synergistic up to 50% growth inhibition	Yang et al^[40]^
Abemaciclib + LY294002		AML (t (8;21)) Abemaciclib (500 nM) + LY249002 (1 µM)	• Induce apoptosis	NA	Nakatani et al^[24]^
Palbociclib + Everolimus/MK-2206 2HCL		AML (FLT3-ITD & FLT3-D835) Pal (0.5 µM) + Everolimus (0.1 µM)	In vitro: • Decrease in cell viability. • Drop in ERK signaling	Bliss Score: Strong Synergy	Uras et al^[28]^
Palbociclib + Everolimus		B-ALL	In vivo: • Combination did not increase CDK4/6 inhibition.	NA	Bride et al^[29]^
Palbociclib + tyrosine kinase inhibitors					
Palbociclib + AURK inhibitors (Danusertib/ Tozasertib/Alisertib/CCT137690)		AML (FLT3-ITD & FLT3-D835Y)	In vitro: • Significant drop in cell viability	Bliss Score: Pronounced Synergy	Uras et al^[28]^
Palbociclib + Imatinib		AML (t(8;21)) Pal:Imatinib (1:10)	In vitro: • Decrease cell viability. • Palbociclib sensitize t(8;21) AML cells to Imatinib	CI: <0.6	Martinez et al^[19]^
Palbociclib + PIM1 inhibitors		AML	In vitro: • Enhance cytotoxicity	Synergistic	Uras et al^[26]^
Palbociclib + Imatinib		ALL (Ph+) Pal (100 nM) + Imatinib (400 nM)	In vitro: • Decrease cell viability	Addictive effect	Nemoto et al^[31]^
		ALL (Ph+) Pal (25 nM) + Imatinib (50 nM)	In vitro: • Decrease cell viability. • Palbociclib sensitize cells to Imatinib	NA	Kuo et al^[41]^
Palbociclib + Ponatinib + Hydroxyurea		CML (T315I + BCR-ABL) Pal (100–400 nM) + Ponatinib (1–4 nM) + HU (25–100 µM)	In vitro: • Inhibit Cell Growth • Palbociclib sensitizes CML cells to Ponatinib. • Triple combination showed the best antiproliferative effect.	Synergistic	Schneeweiss-Gleixner et al^[42]^
Palbociclib + autophagy inhibitors					
Palbociclib + Chloroquine/ 3-MA	AML Pal (25–200 nM) + CQ (25 µM)/ 3-MA (2 mM)		In vitro: • Enhance cytostatic effect of Palbociclib	Synergistic	Gschwind et al^[43]^
Palbociclib + Chloroquine	AML (t (8;21)) Pal (500 nM) + CQ (10 µM)		In vitro: • Suppressed AML cell growth • Induced apoptosis	Synergistic	Nakatani et al^[24]^
Palbociclib + apoptosis promoter					
Palbociclib + Venetoclax + Azacitidine	AML Pal (5 µM) + Ven (0.5 µM) + Aza (1.5 µM)		In vitro: • Promote apoptotic activity in cell lines and primary cells. • Reduce levels of MCL-1 and BCL-X_L_ In vivo: • Decrease in tumor burden in vivo	NA	Wang et al^[11]^
Palbociclib + others					
Palbociclib + GANT1	AML Pal (1 µM) + GANT61 (20 µM)		In vitro: • Palbociclib enhances cytotoxicity of GANT1. • The combination sensitizes cells to AraC.	NA	Zhou et al^[44]^
Palbociclib + BBCG	CLL Pal (10 µM) + BBCG (5–10 µM)		In vitro: • Palbociclib alone decreases GLO1 expression. • BBCG enhance Palbociclib cytotoxicity	NA	Tang et al^[45]^

µM = micromolar, Akt = protein kinase B, ALL = acute lymphoblastic leukemia, AML = acute myeloid leukemia, AraC = cytarabine, Aza = azacitidine, B-ALL = B-cell acute lymphoblastic leukemia, BBCG = S-p-bromobenzyl glutathione cyclopentyl diester, BCL-X_L_ = B-cell lymphoma-extra large, CDK4 = cyclin-dependent kinase 4, CI = combination index, CLL = chronic lymphoblastic leukemia, CML = chronic myeloid leukemia, CQ = chloroquine, Dex = dexamethasone, Dox = doxorubicin, FLT3 = FMS-like tyrosine kinase 3, GFP = green fluorescent protein, GLO1 = glyoxalase 1, IC50 = half-maximal inhibitory concentration, L-asp = L-asparaginase, MCL-1 = myeloid cell leukemia-1, mg/kg = milligrams per kilogram, NA = not applicable, nM = nanomolar, Pal = palbociclib, T-ALL = T-cell acute lymphoblastic leukemia, VCR = vincristine, Ven = venetoclax.

#### 3.2.1. Palbociclib with chemotherapy

Chemotherapy, which involves the use of cytotoxic drugs to target and inhibit the rapid growth of abnormal white blood cells, is the primary treatment for most types of leukemia. While effective in treating cancer, chemotherapy is toxic and may impact healthy cells, leading to side effects. Leukemic cells can also develop resistance to chemotherapy treatment, which requires an increase in doses that could be fatal or completely change the treatment approach. Another approach is to combine chemotherapy with targeted inhibitors, which might help combat the disease by lowering toxicity and increasing effectiveness.

Cytarabine is an antimetabolite commonly used to treat leukemia and lymphoma, which targets the replicating DNA in the S phase.^[46]^ In a study, pretreatment with palbociclib for 24 hours prior to cytarabine induction proved effective in inducing up to 50% cell death in primary AML cells, compared to only 17% when both palbociclib and cytarabine were administered simultaneously.^[22]^ Palbociclib treatment is reversible, which allows cells to enter the S phase simultaneously, thus enhancing the effect of cytarabine.

Palbociclib also induces downregulation of the pathway involving homeobox A9 (HOXA9), PIM1 and B-cell lymphoma (Bcl-2)-associated death promoter (BAD), contributing to the sensitization of AML cells to cytarabine’s apoptotic induction and cytotoxicity.^[22]^ Downregulation of HOXA9 expression resulted in a simultaneous decrease in the expression of the HOXA9-target gene PIM1, which led to the release of inhibition on the phosphorylation of BAD and promoted apoptosis. Notably, the addition of palbociclib prior to cytarabine caused a threefold increase in apoptotic bodies, indicating the favorable effect of the small molecule. Furthermore, administration of low dose of cytarabine (1.6 mg/kg) in combination with palbociclib showed no death in mouse model study, suggesting that palbociclib sensitizes AML cells to cytarabine while allowing safer, reduced dose of cytarabine.^[22]^

Vincristine is a vinca alkaloid and antineoplastic drug that disrupts mitosis, leading to cell cycle arrest and death in rapidly dividing cancer cells.^[47]^ In ALL, palbociclib, in combination with vincristine, appears to be refractory when administered as pretreatment.^[31,39]^ An in vivo experiment observed similar effects when treated with this combination.^[29]^ This non-synergistic effect occurs because vincristine and palbociclib target different cell cycle phases. While palbociclib blocks the progression of the cell cycle from the G1 phase to the S phase, vincristine disrupts cell division by inhibiting microtubule formation that is required during mitosis. However, when palbociclib and vincristine were administered simultaneously, increased apoptotic activity was observed.^[29]^

Palbociclib, combined with other chemotherapy drugs such as dexamethasone, L-asparaginase, and doxorubicin/cytarabine, increases cytotoxic activities and the chemo drugs’ effectiveness in leukemia.^[21,29]^ These indicate the potential use of palbociclib in conjunction with chemotherapy agents as a treatment strategy for leukemia, and several clinical studies are exploring this possibility.^[48–51]^

#### 3.2.2. Palbociclib with targeted therapies

##### 3.2.2.1. FLT3 inhibitors

One of the most common mutations in AML, the FLT3 mutation, occurs in approximately 30% of AML cases and is associated with a poor prognosis.^[37]^ FLT3 inhibitors have emerged as a promising treatment option for AML, effectively targeting and inhibiting the aberrant FLT3 signaling that drives leukemic cell proliferation. However, the treatment effectiveness of FLT3 inhibitors is often limited by the development of resistance.^[27]^ Even though CDK6 is a downstream target of FLT3, it also controls the FLT3 signaling pathways by binding directly to the FLT3 promoter site.^[26]^ This noxious cycle promotes leukemia cell proliferation and survival, making it a critical target for therapeutic intervention. Disrupting this feedback loop by concurrently inhibiting CDK6 and FLT3 offers a synergistic approach to AML treatment.

Deploying this strategy, several studies have shown promising results in in vitro studies.^[26,34,40]^ A study by Wang et al unveiled that palbociclib and SU14813, a FLT3 inhibitor, synergistically enhanced apoptotic activity in FLT3-ITD-positive AML cell lines, MV4-11 and MOLM13, achieving combination indices of 0.78 and 0.67, respectively.^[34]^ Interestingly, this synergism did not extend to primary AML blasts.

Another study by Uras et al revealed that palbociclib exhibits in vitro synergy with TCS-359, a potent FLT3 inhibitor.^[26]^ The combination showed reduced cell growth and increased apoptosis. This effect was similarly demonstrated when palbociclib was combined with tandutinib and quizartinib. In a different experiment, Yang et al demonstrated a synergistic combination of quizartinib and palbociclib.^[40]^ The combination also reduced CDK4 expression and inhibited AKT phosphorylation. Notably, gilteritinib, which is used clinically to treat FLT3-positive AML, was shown to have a more synergistic effect with palbociclib compared to quizartinib.^[40]^ Palbociclib treatment not only enhances the inhibitory effects of FLT3 inhibitors in leukemia cells but also helps to overcome resistance associated with FLT3 inhibitors alone, suggesting targeting both CDK6 and FLT3 could lead to more robust and sustained antileukemic effects.

To summarize, combining palbociclib with FLT3 inhibitors provides a novel treatment approach for AML. This strategy exploits the interaction between CDK6 and FLT3 to improve treatment effectiveness and address resistance challenges, providing a promising development in AML management.

##### 3.2.2.2. PI3K/AKT/MTOR inhibitors

The PI3K/AKT/mechanistic target of rapamycin (mTOR) pathway is a crucial cellular signaling pathway regulating growth, cell survival, and apoptosis. This pathway plays a crucial role in leukemia development and can be a significant target for leukemia therapy. Targeting the PI3K/AKT/mTOR pathway may induce apoptosis and inhibit cell proliferation in hematological malignancies.^[52]^

A study by Yang et al evaluated the combination of palbociclib with several PI3K inhibitors in FLT3-positive AML. All tested P13K inhibitors showed synergistic effects with palbociclib when the growth inhibition was below 50%, with copansilib being the most effective.^[40]^ The combinations shown to reduce the AML-FLT3-positive cell proliferation in a dose-dependent manner and prolonged treatment showed no sign of resistance. The combination also lowers CDK4 expression and inhibits AKT phosphorylation. In an alternate study by Nakatani et al, they assessed a combination of palbociclib with a class III PI3K inhibitor, LY294002, in t(8;21) AML cells, which showed an increase in apoptotic activity.^[24]^ Palbociclib treatment was previously shown to increase autophagy activity in AML, thus inhibition of the class III PI3K, an autophagy upstream regulator, appears effective.

On the other hand, Uras et al showed that palbociclib in combination with the mTOR inhibitor everolimus is synergistic and causes a significant decrease in cell viability in FLT3-positive AML cell lines harboring the D835 mutation in comparison to monotherapy.^[28]^ The combination also caused a decrease in ERK signaling. This was similarly observed when palbociclib was combined with an allosteric AKT inhibitor, MK-2206 2HCl. However, Bride et al demonstrated that the combination of everolimus and palbociclib in vivo did not increase the effect of CDK4/6 inhibition in B-ALL.^[29]^

Targeting the PI3K pathway in combination with palbociclib shows a promising in vitro synergy in AML. However, this only extended in vitro, as the combination failed to show a synergistic effect in vivo.

##### 3.2.2.3. Other tyrosine kinase inhibitors

###### 3.2.2.3.1. Aurora kinase inhibitors

Aurora kinases (AURKs) are a family of serine/threonine kinases with a significant role in cell division. AML cells exhibit atypical expression of AURKs, which are associated with unfavorable cytogenetic abnormalities.^[53,54]^ CDK6 functions as a direct transcriptional regulator for AURK, emphasizing an essential connection between their signaling pathways. Recent research has demonstrated that AURK inhibitors have a strong synergistic effect when combined with palbociclib in AML cells that contain FLT3-ITD and FLT3-D835Y mutations.^[28]^ The observed synergy suggests that the combination of AURK inhibitors and palbociclib has potential as a therapeutic approach for AML treatment.

###### 3.2.2.3.2. Imatinib

Imatinib is a tyrosine kinase inhibitor targeting the Abelson murine leukemia viral oncogene homolog 1 (ABL1), Breakpoint cluster region (BCR)/ABL, platelet-derived growth factor receptor receptor, and KIT proto-oncogene receptor tyrosine kinase (KIT).^[55]^ Some studies have shown that palbociclib can synergize and sensitize leukemic cells to imatinib treatment. For example, Martinez-Soria et al observed combination index of <0.6 for the Kasumi-1 and SKNO-1 cell lines, indicating a significant level of synergy between palbociclib and imatinib.^[19]^ These 2 cell lines express the N882K KIT mutation, which plays a role in AML propagation and is associated with poor clinical outcomes.^[55]^

Additionally, it was discovered that palbociclib, in combination with imatinib, has an additive effect on BCR-ABL and Ph-positive ALL cell lines.^[31,41]^ Studies have demonstrated that palbociclib increases ALL cells’ sensitivity to imatinib, improving its effectiveness. BCR-ABL was shown to increase Cyclin D2 and CDK4 in Ph-positive lymphoid leukemia, explaining the enhanced sensitivity of the cells to palbociclib.^[31]^ These findings emphasize the possibility of improving outcomes in leukemia therapy by combining palbociclib and imatinib.

###### 3.2.2.3.3. PIM1 inhibitors

CDK6 has been linked to regulating FLT3 and PIM1 indicating an intricate interaction in AML.^[26]^ Moreover, PIM1 promotes cell survival and proliferation and engages in the anti-apoptotic pathway through the HOXA9/PIM1/BAD pathway.^[22]^ In FLT3-ITD AML, palbociclib exerts inhibitory effects on CDK6 as well as the FLT3 and PIM1 pathways.^[26]^ The combination of palbociclib with PIM1 inhibitors like SMI-4a has shown synergistic effects, which may provide a therapeutic benefit.^[26]^ Furthermore, the use of palbociclib in combination with dual PIM1/FLT3 inhibitors and SGI-1776 free base demonstrates even greater synergistic effects, suggesting a viable strategy for improving treatment effectiveness through a multifaceted approach in AML.

###### 3.2.2.3.4. Ponatinib

Ponatinib is a potent inhibitor of several tyrosine kinases, effectively targeting diverse mutations, such as T315I in BCR-ABL. Research has indicated that palbociclib increases the sensitivity of CML cells to ponatinib, leading to more potent inhibitory effects on cell proliferation.^[42]^ Furthermore, the combination of palbociclib, ponatinib, and the antimetabolite hydroxyurea has demonstrated the ability to generate more potent cytotoxic effects.

##### 3.2.2.4. Apoptosis promoter

Apoptosis is a natural process of programmed cell death that plays a crucial role in maintaining cellular homeostasis and eliminating damaged or unwanted cells. The upregulation of the B-cell lymphoma 2 (Bcl-2) protein, a key mediator of the mitochondrial apoptotic pathway, correlates with the survival and persistence of AML blasts.^[56]^ Venetoclax binds to Bcl-2 and inhibits it, releasing the pro-apoptotic molecule BAX and promoting mitochondrial-mediated apoptosis.

Recently, venetoclax has been increasingly used in combination with azacitidine (Ven/Aza) as the first-line clinical trial for elderly and frail patients who cannot tolerate the standard treatment.^[57]^ However, one of the expected resistance mechanisms in venetoclax/azacitidine treatment in AML cells is the upregulation of other apoptotic molecules from the Bcl-2 family, MCL-1, and Bcl-xL, to compensate for Bcl-2 loss.^[58]^ Interestingly, the addition of palbociclib to the venetoclax/azacitidine combination caused downregulation of Mcl-1 and Bcl-xL.^[11]^ Palbociclib was shown to promote venetoclax/azacitidine growth inhibition and apoptotic induction activities in KG-1 and THP-1 cell lines. In vivo experiments also showed that palbociclib/venetoclax/azacitidine treatment was effective, as it decreased tumor burden in mice and increased apoptotic activity compared to the venetoclax/azacitidine combination alone. In summary, palbociclib effectively enhances the efficacy of venetoclax/azacitidine treatment both in vitro and in vivo.

##### 3.2.2.5. Autophagy inhibitors

Autophagy is a cellular process that involves the degradation and recycling of cellular components, such as damaged organelles and proteins, within lysosomes. In cancer, neoplastic cells may exploit autophagy as a survival mechanism, allowing them to adapt to environmental stressors, such as nutrient deprivation or drug treatments.^[36]^ Notably, autophagy’s pro-survival function has been shown to contribute to the resistance of leukemia cells to treatment.^[36,59,60]^

Palbociclib treatment was observed to increase autophagy activity in t(8;21) AML cells.^[24]^ Furthermore, it was discovered that combining palbociclib with autophagy inhibitors, LY294002 and chloroquine leads to synergistic induction of apoptosis in primary t(8;21) AML cells, suggesting a promising therapeutic strategy. Extending this research, Matsuo et al explored the efficacy of this combination in vivo, using palbociclib or abemaciclib alongside chloroquine.^[23]^ This study proved the synergistic effect of palbociclib and chloroquine combination to promote leukemic cell apoptosis in vivo. Their findings highlight the potential of exploiting this synergistic interaction between CDK4/6 and autophagy inhibition as a novel approach for targeting AML.

##### 3.2.2.6. Other combinations

Glioma-associated oncogene homolog 1 (GLI1) plays a key role in regulating AML cell proliferation and sensitivity to chemotherapy drugs. GLI1 is an indicator for activating the hedgehog pathway associated with drug resistance in AML, as well as the survival and expansion of BCR-ABL-positive leukemic stem cell.^[61,62]^ GLI1 overexpression caused an increase in the phosphorylation of PI3K and AKT and upregulation of cell cycle regulators GSK3α/β, cyclins D (cyclin D2 and D3), CDK4, and CDK6 that resulted in hyperproliferation and drug resistance in AML.^[44]^ The increase in CDK4 and CDK6 expressions guided the rationale for treating the cells with a combination of the GLI1 inhibitor, GANT61, and the CDK6 inhibitor, palbociclib. The addition of palbociclib reversed the effects of GLI1 overexpression and enhanced GANT61’s apoptotic effect in AML.^[44]^ Moreover, this combination enhanced the sensitivity of AML cell lines and primary cells to cytarabine treatment.

On the other hand, glyoxalase 1 (GLO1) is upregulated in chronic lymphoblastic leukemia and is associated with increased expression of CDK4.^[45]^ Knockdown of CDK4 caused downregulation of GLO1, which is a rate-limiting enzyme of the glyoxalase system, and its overexpression is associated with multiple drug resistances in cancer. Inhibition of GLO1 with S-p-bromobenzyl glutathione cyclopentyl diester appears to reduce cell proliferation and apoptosis and increase cell population in the G0/G1 phase of the cell cycle.^[45]^

## 4. Activity of palbociclib in clinical studies in leukemia

The evidence from clinical trials on the effectiveness of palbociclib in treating leukemia emphasizes its potential to improve treatment approaches. A review on the NCBI’s Clinical Trials database identified ten registered clinical trials, of which the results for 5 of them are provided and summarized in Table 3. The first clinical trial was conducted in 2018 to investigate the impact of palbociclib alone or combined with sorafenib, decitabine, or dexamethasone in relapsed/refractory (R/R) acute leukemia.^[51]^ Palbociclib alone showed limited efficacy, with fatigue, diarrhea, and nausea as the most common adverse events. However, when in combination, there was a marked reduction in extensive extramedullary disease and a decrease in peripheral blasts. Thus, these findings suggest that combination therapies may be more efficacious.

**Table 3:** Palbociclib treatment in clinical trials.

	Clinical Trials					
	Wilde et al^[49]^	Gaidzik et al^[63]^	Raetz et al^[50]^	Nazha et al^[48]^		Kadia et al^[51]^
NCT number	NCT03472573	NCT02310243	NCT03792256	NCT03844997		NCT03132454
Phase	I	Ib/IIa	I	I/II		I
Number ofpatients	7	18	12	14		12
Leukemia subtype	R/R B-ALL	KMT2A-R acute leukemia AML (15) ALL (3)	R/R ALL & L T-ALL (4) B-ALL (7) T-LLy (1)	AML (R/R and newly diagnosed)		R/R Leukemia AML (10) B-ALL (1) T-ALL (1)
Treatment	Palbociclib + Dexamethasone	Palbociclib	Palbociclib + standard 4-drug re-induction chemotherapy	Palbociclib + CPX-351 (Daunorubicin + cytarabine)		Palbociclib + Sorafenib or Decitabine or Dexamethasone
Dose-limiting toxicity (DLT)	0	0	1	0		0
Adverse effect (AE)	G3 Febrile Neutropenia (3 out of 6 patients)	Infection (7) PD (4) Fever (2) Others (8)	Neutropenia (5) Thrombocytopenia (4) Anemia (4) Lymphopenia (3) Sepsis (1) Infections/infestations (1) Catheter-related infection (1) Febrile neutropenia (1) Diarrhea (1) Mucositis (1) Oral pain (1) Hypokalemia (1) Elevated bilirubin (1) Elevated alkaline phosphatase (1)	Febrile neutropenia (6) Elevated bilirubin (1) Epistaxis (1) Electrolyte abnormalities (1) Atrial fibrillation (1) Grade 2 pericarditis/ pericardial effusion (1)		Fatigue (G2) Diarrhea (G2) Nausea (G1)
Response	CRi, relapse (1) PD (5)	CRi (1) PR (1) SD (8) PD (6)	CR/CRi (5) SD: (4) PD: (2)	Phase I: CR (5) Morphological leukemia-free state (1)	Phase II: CR (1) CRp (1) CRi (1)	2 AML + 1 T-ALL = reduced disease burden w/combination therapy

CR = complete remission, CRi = complete remission with incomplete count hematologic recovery, PD = progressive disease, PR = partial remission, SD = stable disease.

Further studies highlighted the potential of palbociclib in combination with chemotherapy in leukemia. Nazha et al investigated the effects of palbociclib in combination with CPX-351 (daunorubicin + cytarabine) in AML patients and reported.^[48]^ Another study showed significant efficacy of palbociclib in combination with routine 4-drug re-induction chemotherapy in R/R ALL, as 5 out of 12 patients achieved CR or incomplete count recovery (CRi).^[50]^ In addition to these findings, Wilde et al reported that 1 R/R B-ALL patient achieved a complete remission with CRi when treated with palbociclib and dexamethasone, emphasizing the potential of this combination.^[49]^ The study, however, was closed earlier due to slow patient accrual.

Finally, Gaidzik et al, in the studies on palbociclib as a stand-alone treatment in ALL and AML, documented that only 1 patient achieved CRi and 1 achieved partial remission (PR) after 2 treatment cycles.^[63]^ In addition, a mere 1 out of 18 patients completed the 6 cycles as outlined in the protocol, while 2 patients died before starting the treatment. Therefore, the findings from this study could be more conclusive. However, the dose-finding phase recommended 125 mg for the expansion phase, according to Gaidzik et al.^[63]^

Regarding safety, the trials found that the adverse effects were controllable and within the anticipated ranges. No dose-limiting toxicity was reported except 1 hematologic dose-limiting toxicity in trials by Raetz et al.^[50]^ Most common toxicities observed were febrile neutropenia, infection, fever, thrombocytopenia, and anemia (Table 3). Kadia et al reported fatigue, diarrhea, and nausea as prevalent side effects associated with palbociclib monotherapy. At the same time, Gaidzik et al stated that the side effects were not solely associated with palbociclib but rather with the overall health status of patients with relapsed or refractory leukemia.^[51,63]^ These studies collectively suggest that the effectiveness of palbociclib as a stand-alone treatment is limited, but it significantly improves and is well tolerated when used in combination therapies.

## 5. Conclusion

Palbociclib is an effective antiproliferative agent in leukemia, even at nanomolar concentrations. The efficacy of palbociclib alone was limited to arresting the cell cycle; however, combined with chemotherapeutic drugs or targeted treatments, it significantly enhanced the treatment outcomes. On the other hand, its antagonistic interactions with other medications, such as vincristine, emphasize the significance of careful treatment choice. Palbociclib is generally well tolerated in clinical settings and has shown promising results, but more evidence is needed. Future studies should identify suitable combination regimens and explore resistance mechanisms, and more extensive clinical trials need to be conducted to validate the preclinical findings, improving patient outcomes for leukemia patients.

## Acknowledgments

We would like to acknowledge the financial support provided by Ministry of Higher Education (MOHE), Malaysia, under the Fundamental Research Grant Scheme (FRGS) grant (FRGS Code: FRGS/1/2022/SKK03/USM/03/1). We also would like to thank Universiti Sains Malaysia for providing the resources and environment necessary for the completion of this review.

## Author contributions

**Conceptualization:** Muhamad Amir Azizan.

**Investigation:** Muhamad Amir Azizan, Zainul Abeden.

**Supervision:** Mohd Nazri Ismail, Siew Kit Ng, Asmida Isa.

**Validation:** Fadly Ahid, Narazah Mohd Yusoff, Mohd Nazri Ismail, Siew Kit Ng, Asmida Isa.

**Writing – original draft:** Muhamad Amir Azizan, Zainul Abeden, Nur Haida Natasha Shamsuddin.

**Writing – review & editing:** Muhamad Amir Azizan, Zainul Abeden, Nur Haida Natasha Shamsuddin, Fadly Ahid, Narazah Mohd Yusoff, Asmida Isa.
